# Chronic inflammation up-regulates P-gp in peripheral mononuclear blood cells via the STAT3/Nf-κb pathway in 2,4,6-trinitrobenzene sulfonic acid-induced colitis mice

**DOI:** 10.1038/srep13558

**Published:** 2015-09-01

**Authors:** Jiali Liu, Fang Zhou, Qianying Chen, An Kang, Meng Lu, Wenyue Liu, Xiaojie Zang, Guangji Wang, Jingwei Zhang

**Affiliations:** 1 Key Lab of Drug Metabolism and Pharmacokinetics, State Key Laboratory of Natural Medicines, China Pharmaceutical University, Nanjing, Jiangsu, China; 2 Jiangsu Key laboratory of drug design and optimization, China Pharmaceutical University, Nanjing, Jiangsu, China; 3 School of Pharmacy, Nanjing University of Chinese Medicine, Nanjing, Jiangsu, China

## Abstract

Patients with inflammatory bowel diseases, including Crohn’s disease and ulcerative colitis, often suffer drug intolerance. This resistance can be divided into intrinsic resistance and acquired resistance. Although there is agreement on acquired resistance, studies regarding intrinsic resistance have demonstrated inconsistencies, especially for Crohn’s disease. For this reason, an animal model of Crohn’s disease was induced with 2,4,6-trinitrobenzene sulfonic acid solution (TNBS), and intrinsic resistance was analyzed by measuring the function and expression of P-glycoprotein (P-gp) in peripheral mononuclear blood cells (PMBC), followed by mechanistic studies. The results revealed reduced retention of cyclosporine A in PMBC over-expressing P-gp in a TNBS-treated group and enhanced secretion of the cytokines IL-1β, IL-6, IL-17, and TNF-α as well as LPS in plasma. These cytokines and LPS can induce P-gp expression through the STAT3/Nf-κb pathway, contributing to a decrease of cyclosporine A retention, which can be reversed by the application of a P-gp inhibitor. Our results demonstrated that the sustained chronic inflammation could induce the intrinsic resistance presented as P-gp over-expression in PBMC in Crohn’s disease through STAT3/Nf-κb pathway and this resistance might be reversed by combinational usage of P-gp inhibitors.

Inflammatory bowel disease (IBD) is defined as chronic intestinal inflammation and mainly includes Crohn’s disease (CD) and ulcerative colitis (UC). Statistics indicate that IBD affects almost 3.6 million people in Europe and the US every year, and the number of IBD patients in Asia is also rapidly increasing^1^,^2^. Until now, many causes have been reported to contribute to the development of IBD, such as dietary habits, environmental factors, genetic susceptibility and infectious microbes, but the exact mechanism of this disease is still unknown^3^,^4^. Because IBD patients exhibit significantly dysregulated immune systems, IBD is also currently characterized as an autoimmune disease^5^,^6^. Hence, immunosuppressive drugs and anti-inflammatory agents are commonly involved in the clinical treatment of IBD with the purpose of diminishing symptoms and decreasing inflammation in the colon lining^7^,^8^,^9^.

However, remission and drug intolerance always occur during drug treatment of autoimmune disease, which causes a compromising effect^10^. It has been widely reported that a failure to respond to glucocorticoid therapy is a common indication of treatment for IBD, as well as of treatment for systemic lupus erythematous and other autoimmune diseases^11^,^12^,^13^,^14^,^15^. This resistance can be divided into two categories, intrinsic resistance and acquired resistance, and both are highly related to the increased expression of the efflux pump P-glycoprotein (P-gp) in peripheral mononuclear blood cells (PMBC). In intrinsic resistance, patients exhibit little or no sensitivity to immunosuppressants when they begin taking medication. Because the over-production of pro-inflammatory cytokines that occurs in autoimmune disease can induce the expression of P-gp, which pumps immunosuppressive drugs out of PBMC and thus attenuates drug efficacy^16^,^17^. Conversely, in acquired resistance, patients gradually show resistance to immunosuppressants after long-term drug treatment, as immunosuppressants themselves can induce the expression of P-gp in immune cells during usage^18^,^19^. For example, the expression of P-gp in the PBMC of UC patients undergoing glucocorticoid (GC) therapy was significantly greater than in those not undergoing GC administration^20^.

Even though there is agreement about the concept of acquired resistance in patients with IBD, studies regarding intrinsic resistance have demonstrated inconsistencies. Bruce reported greater and differential expression and functioning of P-gp in patients with CD compared to those with UC or those with normal tissues, while results from Toshihiko indicated similar amounts of P-gp expression in the PBMC of healthy subjects and CD patients or in UC patients not undergoing GC therapy^20^,^21^. Furthermore, the reasons behind the drug resistance and change in P-gp expression in patients with CD are still unclear. In acquired and intrinsic resistance during cancer treatment, an increased expression of P-gp has been reported to result from the activation of NF-κB^22^,^23^. This transcription factor, which can promote pro-inflammatory responses, also demonstrates sustained activation in patients with colitis and has been reported to be a key target of many of the drugs that are used for IBD treatment, such as ginsenosides and DIMSO150^24^,^25^. STAT3 is highly associated and demonstrates crosstalk with NF-κB activation^26^,^27^ and also plays an important role in IBD^28^. A number of studies using mouse models have suggested that epithelial Stat3 activation is essential for the maintenance of gastrointestinal barrier integrity, and cell populations in which STAT3 is constitutively active may dictate the pathology of IBD^29^,^30^. Even though a relationship between STAT3/NF-κB and IBD has been demonstrated, it remains unclear whether or how it functionally contributes to P-gp expression and the kinetic profile of drug treatment in IBD. Therefore, our research is focused on evaluating intrinsic resistance in CD through P-gp regulation from a pharmacokinetics viewpoint by using a trinitrobenzene sulfonic acid (TNBS)-induced murine model, which well describes the characteristics of human CD patients^31^.

## Materials and Methods

### Ethics Statement

All animal experiments were approved by the Animal Ethics Committee of China Pharmaceutical University (Nanjing, China). This study was carried out in strict accordance with the Guidelines for Animal Experimentation of this institution. All procedures were performed as humanely as possible. Every effort was made to minimize animal pain, suffering and distress and to reduce the number of animals being used.

### Chemicals and reagents

2,4,6-Trinitrobenzene sulfonic acid solution (TNBS), cyclosporine A (Cys A), Rhodamine 123 (Rho 123), FK506, LY 335979, Stattic, Bay 117082, methanol and acetonitrile were all purchased from Sigma-Aldrich (St. Louis, MO, USA). Human IL-6, IL-1β, TNF-α, IL-17 and LPS were all purchased from Peprotech (Rocky Hill, USA). APC-conjugated anti-mouse CD3, CD14, CD19 antibodies were purchased from Biolegend (San Diego, CA, USA). FITC-conjugated anti-mouse P-gp antibody was purchased from Beijing Biosynthesis Biotechnology Co., Ltd (Beijing, China). FITC-conjugated anti-human P-gp antibody was purchased from BD Bioscience (San Jose, CA, USA). Monoclonal antibodies against stat3, p-stat3, p65, p-p65, lamin B, and β-actin and horseradish peroxidase-conjugated goat anti-mouse/rabbit IgG secondary antibodies were purchased from Cell Signaling Technology (Danvers, MA, USA). A SYBR Prime Script RT-PCR Kit was purchased from Takara Bio Inc. (Otsu, Shiga, Japan). Deionized water was prepared using a Milli-Q system (Millipore, Milford, MA, USA) and was used throughout the study.

### Animals

Healthy male Wister rats (180–220 g and 8–10 weeks) and BALB/c mice (18–22 g and 8–10 weeks) were obtained from Shanghai SLAC Laboratory Animal Co. Ltd (Shanghai, China) and were housed ten per cage at room temperature (22±1 °C) with 50–60% relative humidity and an automatic day-night rhythm (12 h-cycle) in a clean-grade environment. Prior to each experiment, the animals were fasted overnight (12 h) with free access to water.

### TNBS-induced colitis

Briefly, the rats/mice were anesthetized via intraperitoneal injection of pentobarbital (80 mg/kg). Then, the rats/mice were placed in a head-down position and slowly administered TNBS (20 mg/0.25 ml/0.2 kg in 30% ethanol for rats and 2.5 mg/0.1 ml/0.02 kg in 50% ethanol for mice) into the colon via a thin catheter. Meanwhile, the control groups received a vehicle (30% ethanol for rats or 50% ethanol for mice). After cannula withdrawal, the rats/mice were maintained in the position for 3 min to avoid leakage of the intracolon instillation and were then kept on warm bedding until they regained consciousness. The rats/mice in both the model group and the control group were kept for 3 or 7 days, and the body weight of each mouse was recorded daily. At designated times, peripheral blood was collected via the retro-orbital venous sinus into heparinized tubes, and the rats/mice were sacrificed by CO_2_ asphyxiation and cervical dislocation. The proximal colons of the mice were collected, measured, and imaged immediately.

### Pharmacokinetics assay

On the 7^th^ day, the rats in the control group and the TNBS-induced model group were intravenously administered with Cys A at a dosage of 10 mg/kg. Post drug administration, blood was collected at 0.08, 0.5, 1, 2, and 4 h via the retro-orbital venous sinus into heparinized tubes, further separated into plasma and PBMC, and stored at −20 °C before analysis. Concentrations of Cys A were determined by LC-MS/MS.

### Histology Assays

Specimens of mouse proximal colon (2.5 cm) were fixed in 10% neutral-buffered formalin for 24 h and embedded in paraffin. Sections were cut at a thickness of 5 μm, stained with H&E, and imaged with Leica QWin software (Leica Microsystems, Bensheim, Germany). Histological damage was evaluated and graded in a double-blinded fashion by a skilled pathologist according to previously described criteria.

### Determination of cytokine and LPS in plasma

The concentrations of the cytokines IL-1β, IL-6, IL-17 and TNF-α were determined in mouse plasma using commercially available ELISA kits (Excell, Shanghai, China), of which the lower limit of quantification was 10 pg/ml for each cytokine. LPS was measured using a Limulus Amebocyte Lysate (LAL) assay kit (Xiamen Hou Reagent Company, Fujian, China) according to the manufacturer’s protocol. The lower level of detection for this assay was 0.7 pg/ml. All of the samples were run in triplicate, and the mean values are reported.

### Isolation of peripheral blood mononuclear cells

Peripheral blood mononuclear cells (PBMC) were isolated by density gradient centrifugation over Lympholyte Mammal (Cedarlane Laboratories Ltd, Hunby, Ontario, Canada) according to the instructions of the manufacturer. Briefly, blood was diluted 1:1 with RPMI-1640 medium (v/v), precisely applied on the surface of the gradient (density: 1.086 ± 0.001 g/cm^3^) and centrifuged at 800 × g for 20 min. Then, the PBMC layer at the interface was carefully collected and transferred into a 15-ml Falcon tube, washed with RPMI-1640 medium and centrifuged again for 10 min at 800 × g. The obtained cell pellet was re-suspended in RPMI-1640 medium for further experiments.

### Flow cytometry analysis of PBMC subsets with P-gp positive expression

Isolated PBMC were incubated with FITC-conjugated anti-mouse P-gp antibody together with APC-conjugated anti-mouse CD3, CD14 or CD19 antibodies. Data were acquired on a BD FACSVerse (Becton and Dickinson, NJ, USA) and then analyzed. Briefly, cells were first gated for lymphocytes or monocytes using SSC-H vs. FSC-H, and then FITC/APC double-positive staining was considered to represent positive P-gp expression in T cells (CD3^+^), B cells (CD19^+^) and/or monocytes (CD14^+^).

### Cell Culture

The human T lymphoblast CCRF-CEM cell line and the human monocyte THP-1 cell line were purchased from American Type Culture Collection. The CCRF-CEM MDR1 cell line was constructed by GenScript Co., Ltd. (Nanjing, China) from its parent cell line CCRF-CEM by lentiviral transfection; it overexpressed human P-gp. All of the cells were grown as suspension cultures in RPMI-1640 medium supplemented with 10% fetal bovine serum and 100 U·ml^−1^ penicillin and streptomycin (Gibco-Invitrogen, USA). The cells were grown in an atmosphere of 5% CO_2_ at 37 °C, and the cell medium was changed every 2 to 3 days.

### P-gp Expression Assay

The cells were fixed with a 4% paraformaldehyde solution, followed by washing and blocking. Then, the cells were incubated with a FITC-conjugated anti-human P-gp antibody or an isotype-matched negative control for 1 h at 37 °C. After washing, the cells were analyzed by flow cytometry (BD FACSVerse, NJ, USA) for P-gp protein expression.

### Quantitative Real-time PCR

Total RNA was extracted from the cells using Trizol reagent (Takara, Kyoto, Japan) and was reverse-transcribed into cDNA using a Prime Script RT Reagent Kit (Takara, Kyoto, Japan). qPCR reactions were performed in a CFX96 Real-Time PCR Detection System (Bio-Rad, CA, USA) using SYBR Premix Ex Taq II (Takara, Kyoto, Japan). The sequences of each gene are as follows:

Human-mdr1-F 5′ GCTGGGAAGATCGCTACTGA 3′,

Human-mdr1-R 5′ GGTACCTGCAAACTCTGAGCA 3′,

Human-actin-F 5′ GCGTGACATTAAGGAGAAG 3′,

Human-actin-R 5′ GAAGGAAGGCTGGAAGAG 3′,

Mouse-mdr1a-F 5′ AGGGCATTTACTTCAAACTTGTC 3′,

Mouse-mdr1a -R 5′ CCTGTCTTGGTCATGTGGTC 3′,

Mouse-actin-F 5′ TCTGGCACCACACCTTCTA 3′,

Mouse-actin-R 5′AGGCATACAGGGACAGCAC 3′.

The qPCR conditions included 95 °C for 90 seconds, followed by 40 cycles of 95 °C for 10 seconds, 60 °C for 30 seconds, and 72 °C for 30 seconds. Primer specificity was monitored using product melting curves in each reaction well.

### Cellular Retention Assay

Briefly, the cells were incubated in RPMI-1640 medium containing drugs in the absence or presence of LY335979 (P-gp inhibitor) at 37 °C for the designated time. The accumulation was stopped by rinsing the cells with blank medium. Cells were then lysed by three freeze-thaw cycles, and protein concentrations were measured by the Bradford method using a BCA protein assay kit (Thermo Fisher Scientific, Waltham, MA, USA). Rho 123 was assayed as we described previously^32^. The concentration of Cys A was determined by LC-MS/MS. All experiments were conducted in triplicate.

### LC-MS/MS Analysis

Briefly, a sample aliquot of 100 μl was protein-precipitated with 300 μl methanol. After centrifugation, 10 μl of the supernatant was injected into an AB SCIEX API-4000 tandem mass spectrometer (Foster City, CA, USA) equipped with electrospray ionization (ESI), LC-20AD pumps, a SIL-20A auto-sampler, and a CTO-20A oven (Shimadzu, Kyoto, Japan). Analyst 1.5.1 software was used for data acquisition and processing. Separations were conducted on a Luna C8 column (100 × 2.0 mm, 5.0 μm; Phenomenex Inc, USA), and the column temperature was maintained at 40 °C. A binary gradient consisting of solvent A (0.1% formic acid solution with 5 mM ammonium acetate) and solvent B (methanol) was employed for the elution of Cys A and FK506 (internal standard). The flow rate was kept at 0.45 ml·min^−1^ by the following gradient program: 0–1.0 min, 75% B; 1.0–1.5 min, 75–99.5% B; 1.5–4.5 min, 99.5% B; 4.5–5.0 min, 99.5–75% B; 5.0–10.0 min, 75% B. The parameters of ESI source in positive mode were optimized as follows: curtain gas, 30 Arb; Gas 1, 65 Arb and Gas 2, 65 Arb; source temperature, 500 °C; ionization voltage, 5500 V. The mass spectrometer was operated in multiple reaction monitoring (MRM) mode (m/z 1224.9 → 1112.8 for Cys A, m/z 821.4 → 768.3 for FK506).

### Western Blot Assay

For Western blot analysis, nuclear, cytoplasmic and whole cell extracts were prepared as described previously^33^,^34^. The protein samples were separated on a 10% SDS-polyacrylamide gel and transferred onto a polyvinylidene difluoride membrane (Bio-Rad, CA, USA). The membrane was blocked with 5% bovine serum albumin, followed by incubation with primary antibodies and a horseradish peroxidase- conjugated secondary antibody. Antibody signals were detected using an enhanced chemiluminescence kit (Thermo Fisher Scientific, Waltham, MA, USA) and were captured using a ChemiDoc XRS^+^ System (Bio-Rad, CA, USA). All blots were stripped and probed with anti-actin or anti-lamin B antibodies to ascertain equal loading of the proteins.

### Data Analysis

Data are expressed as the mean ± S.E.M. Statistical analysis was performed using a two-tailed Student’s *t*-test and one-way ANOVA. Differences were considered to be statistically significant if the probability value was less than 0.05 (p < 0.05).

## Results

### Decreased plasma and cellular concentrations of Cys A in TNBS-induced rats

**Figure 1:**
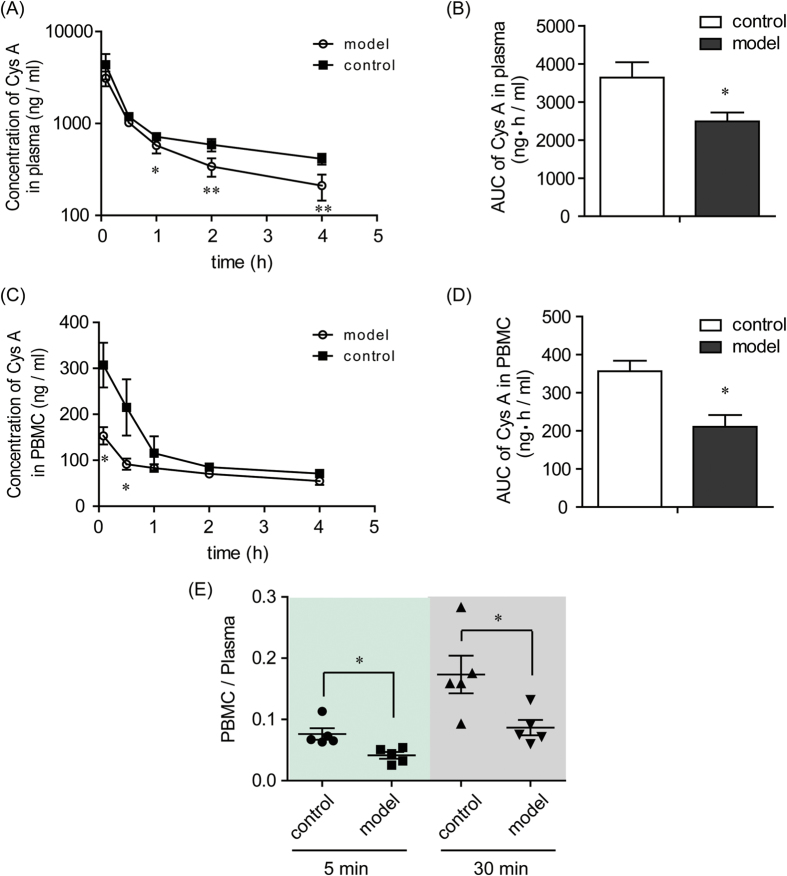
Plasma and cellular pharmacokinetic profiles of Cys A in control and TNBS-induced rats. The control group was given a blank vehicle intrarectally, and the model group was administered a dose of 100 mg/kg TNBS intrarectally. On the 7^th^ day, both groups were intravenously administered Cys A at a dosage of 10 mg/kg. Blood was collected at the designated time points post Cys A administration, and plasma and PBMC were separated. Concentrations of Cys A in plasma and PBMC were determined by LC-MS/MS, and concentration-time curves of Cys A in plasma (**A**) and PBMC (**C**) were plotted. The AUC of Cys A in plasma (**B**) and in PBMC (**D**) was calculated using the trapezoidal method. The ratio of Cys A concentration in PBMC to that in plasma at 5 min and 30 min post Cys A administration was also calculated (**E**). Data are expressed as the mean ± S.E.M.; n = 5/group. *p < 0.05, **p < 0.01 between the model group versus the corresponding control group.

Compared with the rats in the control group, the concentrations of Cys A in the plasma of TNBS-induced rats (model group) at 5 min and 30 min post drug administration were not significantly changed, while they were markedly decreased at the 1 h, 2 h and 4 h time points (Fig. 1A). The AUC of Cys A was decreased from 3637.66 ± 404.95 ng·h/ml in the control group to 2489.99 ± 232.29 ng·h/ml in the model group (Fig. 1B).

The concentrations of Cys A in the PBMC of the model groups decreased markedly at the 5 min and 30 min time points when compared with the control group, although they were unchanged at later time points (Fig. 1C). The AUC of Cys A in the PBMC also decreased from 356.39 ± 27.51 ng·h/ml in the control group to 210.42 ± 30.98 ng·h/ml in the model group (Fig. 1D). The ratio of drug concentrations in PBMC to those in plasma at the 5 min and 30 min time points in the model group was significantly decreased and was only approximately half the value of the ratio in the control group (Fig. 1E).

### Increased expression and function of P-gp in the PBMC of TNBS-induced mice

**Figure 2:**
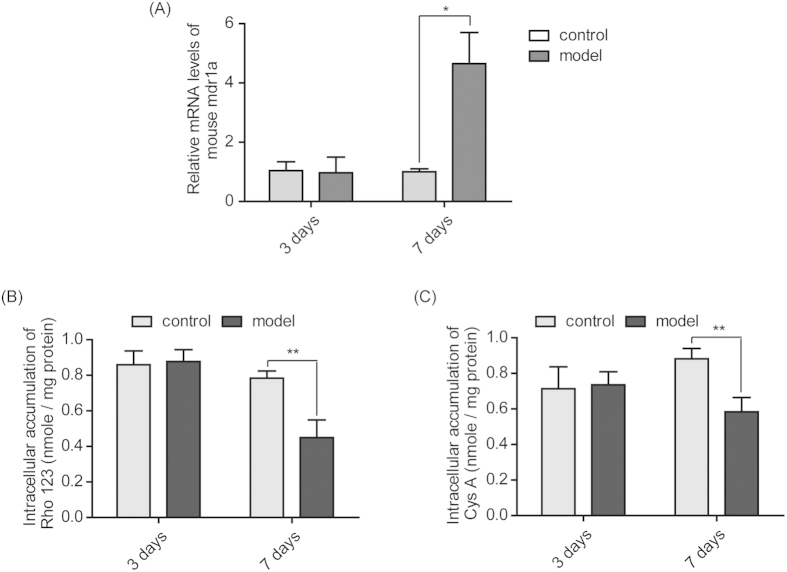
Elevated expression and function of P-gp in the PBMC of TNBS-induced mice. PBMC were isolated from the peripheral blood of mice in the control group and the TNBS-treated model group on the 3^rd^ and 7^th^ days post vehicle or TNBS challenge. The gene expression of mdr1a in the PMBC was analyzed by qPCR (**A**). The function of P-gp in the PBMC was assayed by incubating the PBMC with the P-gp substrate probe Rho 123 (**B**) or the P-gp substrate immunosuppressant Cys A (**C**) for 2 h to determine its intracellular retention. Data are presented as the mean ± S.E.M., *p<0.05, **p<0.01 between the model group versus the corresponding control group; n = 8/group.

Model mice were successfully treated with TNBS and kept for 3 days (acute model) and 7 days (subacute model) (Supplementary Fig 1). As shown in Fig 2A, the expression of the mdr1a gene in PBMC isolated from model mice treated with TNBS for 3 days exhibited no difference from the control groups. However, when these model mice were treated with TNBS for 7 days, mdr1a gene expression in the PBMC of the model group was significantly elevated to approximately 4.65-fold of that in the control group (p < 0.05).

When these isolated PBMC were incubated with the P-gp substrates Rho 123 or Cys A, there was no difference in intracellular accumulations between the control group and the model group treated with TNBS for 3 days. However, for the 7-day TNBS-induced mice, the intracellular accumulations of Rho 123 and Cys A in PBMC were significantly decreased, being only 57.3% or 65.9% of the corresponding control groups (Fig. 2B,C).

### T-lymphocytes with higher P-gp expression dominated in the PBMC of TNBS-induced mice

**Figure 3:**
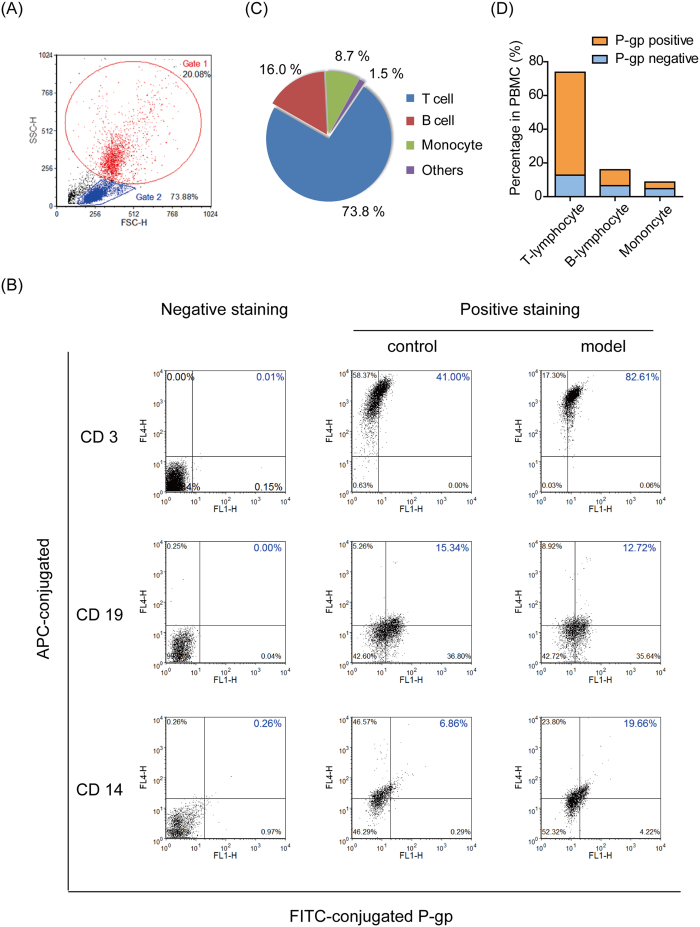
T-lymphocytes with high P-gp expression dominated the PBMC of TNBS-induced mice. Isolated PBMC were incubated with FITC-conjugated anti-mouse P-gp antibody together with APC-conjugated anti-mouse CD3, CD14 or CD19 antibodies. The data were acquired on a BD FACSVerse flow cytometer and analyzed. (**A**) The cells were first gated crudely for monocytes (Gate1) or lymphocytes (Gate2) using SSC-H vs. FSC-H. (**B**) The cells in Gate1 or Gate2 were further quadrant-gated by comparing them with the negative-stained group, and FITC/APC double-positive staining (upper right in quadrant gating) was considered to represent positive P-gp expression in T cells (CD3^+^), B cells (CD19^+^) and monocytes (CD14^+^). (**C**) By multiplying the percentage of APC-positive (upper left and upper right quadrant gating) cells in Gate1/Gate2 by the percentage of Gate1/Gate2 in PBMC, the relative contents of T-lymphocytes, B-lymphocytes and monocytes in the PBMC of colitis mice were determined. (**D**) By multiplying the percentage of FITC/APC double-positive (upper right in quadrant gating) cells in Gate1/Gate2 by the percentage of Gate1/Gate2 in PBMC, the percentages of positive P-gp expressing T-lymphocytes, B-lymphocytes and monocytes in the PBMC of colitis mice were determined.

As seen in Fig 3A, PBMC were gated crudely for the monocyte group (Gate 1, 20.08%) and the lymphocyte group (Gate 2, 73.88%) by using SSC-H vs. FSC-H. Then, the cells in each gate were further identified by APC/FITC double-staining to certify P-gp positive expression in a subset of PBMC. Focusing on the upper right quadrant gating in Fig 3B, the percentages of positive P-gp-expressing T-lymphocytes (CD3^+^) and monocytes (CD14^+^) were increased significantly from 41.0% to 82.6% and from 6.8% to 19.6%, respectively, when colitis was induced in normal mice. However, the percentage of P-gp positive expression in B-lymphocytes (CD19^+^) was not changed markedly.

Compared with the negative-staining group, APC-conjugated CD3 was strongly positive with a significant up-shift in quadrant gating (upper left and upper right), while APC-conjugated CD19 and CD14 were only weakly positive with a minor up-shift. By further multiplying the percentage of the APC-positive cells in the Gate by the percentage of the Gate in PBMC, the relative contents of T-lymphocytes, B-lymphocyte and monocytes in the PBMC of colitis mice were found to be 73.8%, 16.0% and 8.7%, respectively (Fig. 3C), of which positive P-gp expression was observed in T-lymphocytes, B-lymphocytes and monocytes at proportions of 61.0%, 9.4% and 3.9%, respectively (Fig. 3D).

### Increased cytokine and LPS levels in peripheral plasma of TNBS-induced mice

**Figure 4:**
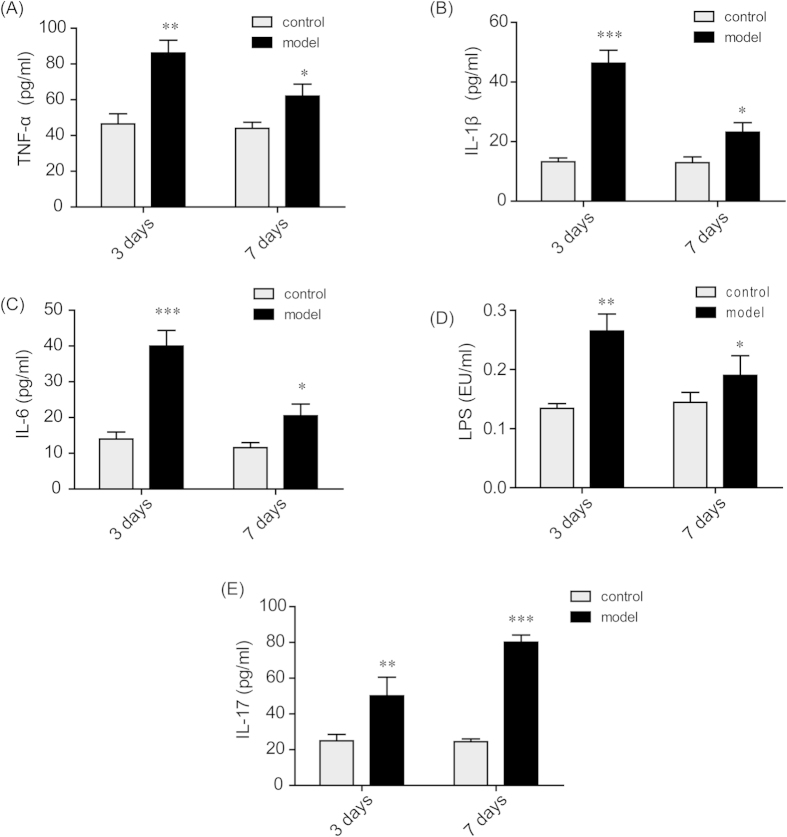
Increased cytokine and LPS levels in the plasma of TNBS-induced mice. Plasma samples from the mice in the control group and the TNBS-treated model group were collected on the 3^rd^ and 7^th^ day after vehicle or TNBS administration. The concentrations of the cytokines TNF-α (**A**), IL-1β (**B**), IL-6 (**C**) and IL-17 (**E**) were determined by ELISA kits. The level of LPS (**D**) was analyzed by a Limulus Amebocyte Lysate assay. The data are presented as the mean ± S.E.M.; *p < 0.05, **p < 0.01, ***p < 0.001 between the model group versus the corresponding control group; n = 8/group.

As seen in Fig 4, the levels of LPS and the cytokines TNF-α, IL-1β, IL-6, and IL-17 in peripheral plasma were boosted sharply when model mice were treated with TNBS for 3 days. When model mice were challenged with TNBS for 7 days, the levels of LPS and the cytokines TNF-α, IL-1β, and IL-6 were still significantly higher than those of the control group, but they were markedly lower than those of the 3-day group. However, the level of IL-17 increased continuously throughout the 7 days post TNBS challenge, and the level of the 7-day group was significantly higher than that of 3-day group.

### Cytokines and LPS increased MDR1 mRNA expression in T-lymphocytes and monocytes *in vitro*

**Figure 5:**
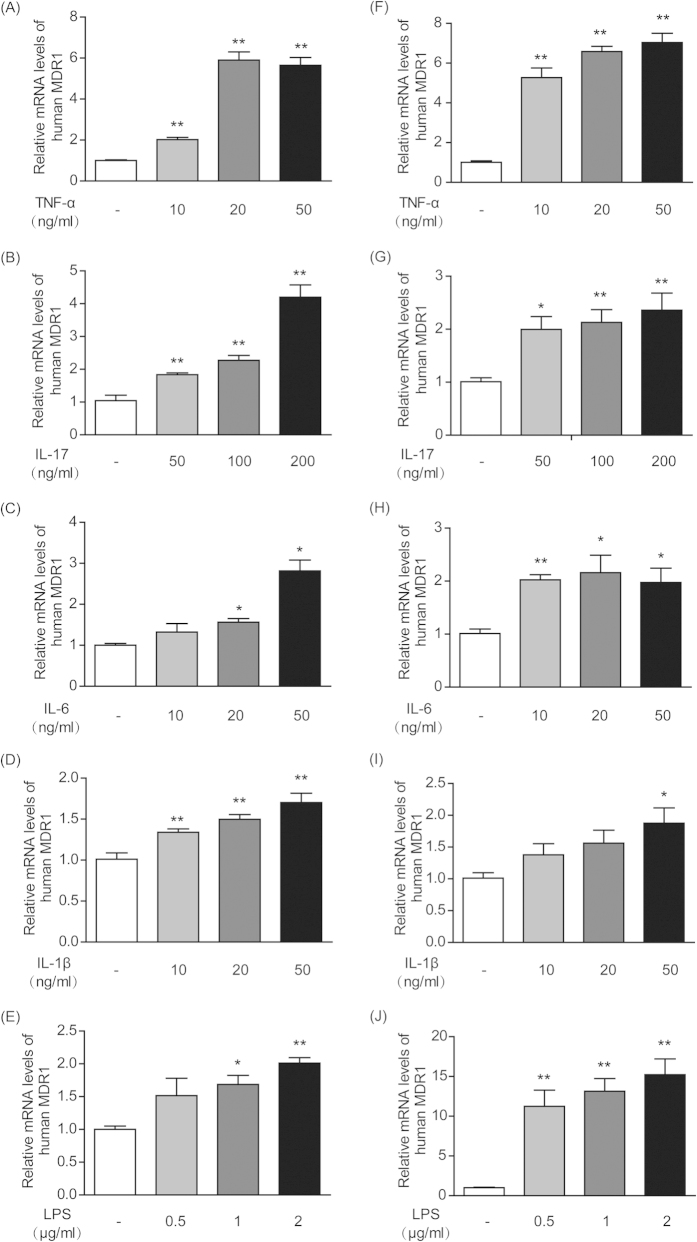
Cytokines and LPS increased MDR1 gene expression in human T-lymphocytes and monocytes *in vitro*. CCRF-CEM cells were cultured with different concentrations of TNF-α (**A**), IL-17 (B), IL-6 (**C**), IL-1β (**D**) and LPS (**E**). THP-1 cells were also incubated with various concentrations of TNF-α (**F**), IL-17 (**G**), IL-6 (**H**), IL-1β (**I**) and LPS (**J**). The mRNA levels of MDR1 in the CCRF-CEM and THP-1 cells were measured by qPCR. Actin was used as an internal control. All of the data are shown as the mean ± S.E.M.; *p < 0.05, **p < 0.01 compared with the corresponding control group; n = 3.

As shown in Fig 5, when human T-lymphocyte CCRF-CEM cells and human monocyte THP-1 cells were incubated with LPS and the cytokines TNF-α, IL-1β, IL-6, and IL-17, then MDR1 gene expression was up-regulated in a concentration-dependent manner to different extents. In CCRF-CEM T-lymphocyte cells, the cytokines TNF-α and IL-17 exhibited the most potent stimulatory effects and could increase MDR1 expression by up to 5.9-fold (Fig. 5A) and 4.2-fold (Fig. 5B), respectively. In THP-1 monocyte cells, LPS presented the most potent effect and elevated MDR1 expression by 15.2-fold (Fig. 5J). Next in importance was TNF-α, which could raise MDR1 expression by 7.1-fold (Fig. 5F).

**Figure 6:**
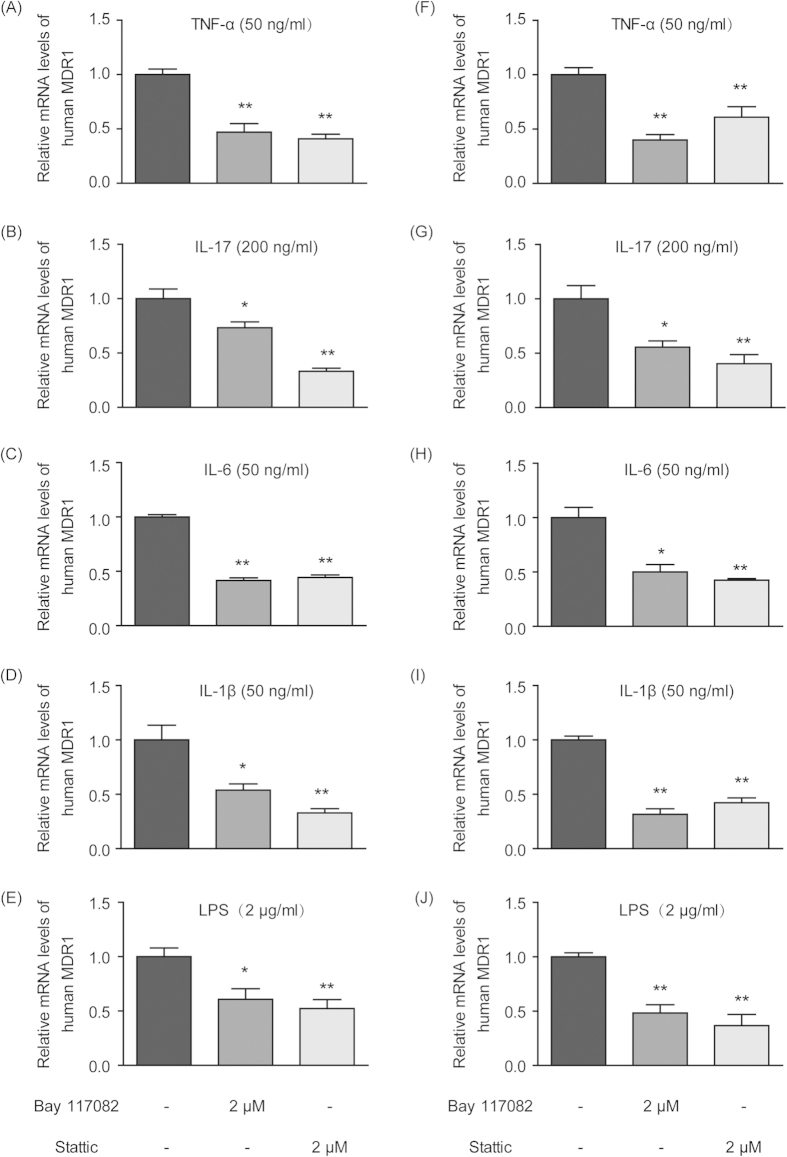
Nf-κb and STAT3 inhibitors attenuated cytokine- and LPS-induced elevations in MDR1 gene expression in human T-lymphocytes and monocytes *in vitro*. CCRF-CEM cells were cultured with 50 ng/ml TNF-α (**A**), 200 ng/ml IL-17 (**B**), 50 ng/ml IL-6 (**C**), 50 ng/ml IL-1β (**D**) and 2 μg/ml LPS (**E**) in the absence or presence of the Nf-κb inhibitor Bay 117082 (2 μM) and the STAT3 inhibitor Stattic (2 μM). THP-1 cells were also incubated with 50 ng/ml TNF-α (**F**), 200 ng/ml IL-17 (**G**), 50 ng/ml IL-6 (H), 50 ng/ml IL-1β (**I**) and 2 μg/ml LPS (**J**) with or without the Nf-κb inhibitor Bay 117082 (2 μM) and the STAT3 inhibitor Stattic (2 μM). The mRNA levels of MDR1 in the CCRF-CEM and THP-1 cells were measured by qPCR. Actin was used as an internal control. All of the data are shown as the mean ± S.E.M.; *p < 0.05, **p < 0.01 compared with the corresponding control group; n = 3.

When CCRF-CEM T-lymphocyte cells and THP-1 monocyte cells were treated with pro-inflammatory cytokines (TNF-α, IL-1β, IL-17 and IL-6) and LPS in the presence of the Nf-κb inhibitor Bay 117082 or the Stat3 inhibitor Stattic, the originally elevated MDR1 expression was significantly down-regulated to different extents (Fig. 6), suggesting the potential involvement of the STAT3/Nf-κb pathway.

### IL-17 increased P-gp expression through the STAT3-dependent Nf-κb pathway

**Figure 7:**
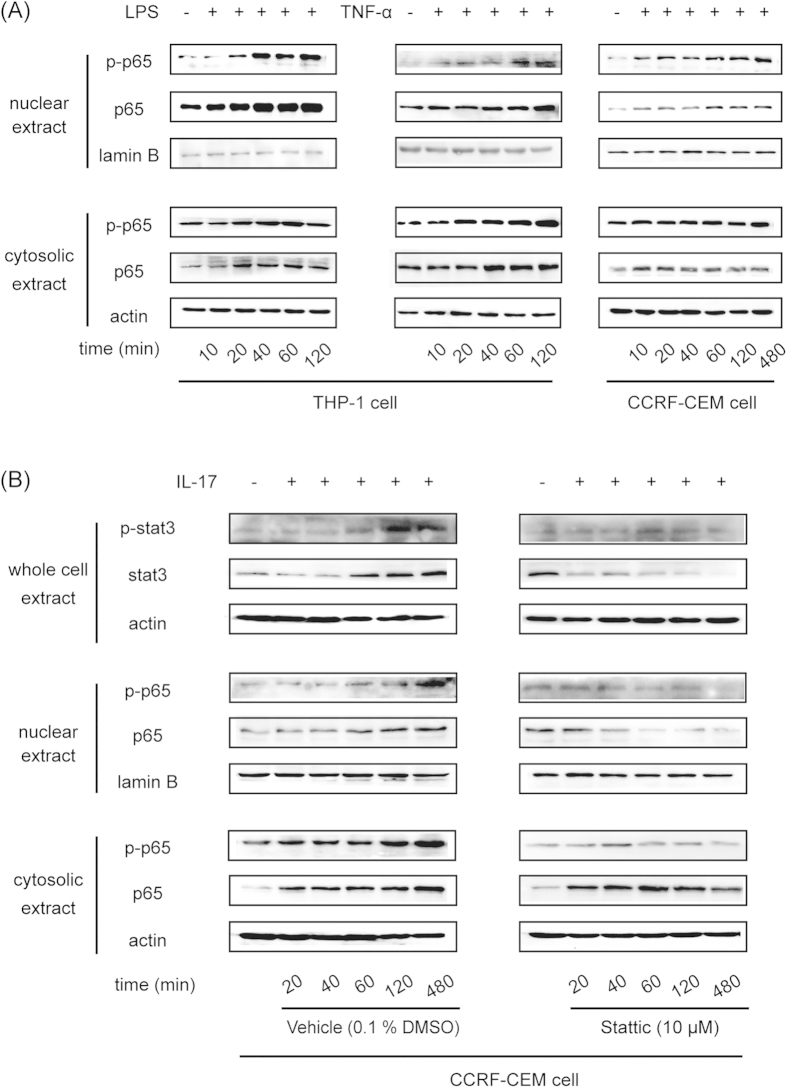
IL-17 increased P-gp expression through the STAT3-dependent Nf-κb pathway. (**A**) THP-1 cells were treated with 50 ng/ml TNF-α or 2 μg/ml LPS for different periods of time. CCRF-CEM cells were treated with 50 ng/ml TNF-α for different periods of time. Nuclear and cytosolic p65 and phosphorylated p65 were detected by Western blot. (**B**) CCRF-CEM cells were treated with 200 ng/ml IL-17 in the absence or presence of the STAT3 inhibitor Stattic for different periods of time. Total STAT3 and phosphorylated STAT3 and nuclear and cytosolic p65 and phosphorylated p65 were detected by Western blot.

LPS and the cytokine TNF-α could stimulate both p65 and phosphorylated p65 expression in the cytosol and nuclei of THP-1 cells in a time-dependent manner (Fig. 7A). In CCRF-CEM cells, TNF-α promoted the translocation of p65 from cytosol to nuclei, with decreased p65 expression in the cytosol and increased p65 expression in the nuclei. Furthermore, TNF-α also enhanced the phosphorylation of p65 in the nuclei in a time-dependent manner (Fig. 7A).

IL-17 increased STAT3 expression and augmented the phosphorylation of STAT3 with prolonged incubation times. Meanwhile, IL-17 promoted the expression and phosphorylation of p65 both in cytosol and nuclei. When the STAT3 inhibitor Stattic was used in combination with IL-17, all of the above-mentioned effects of IL-17 were weakened or even reversed. Stattic decreased the expression of STAT3 and prevented its phosphorylation. Furthermore, Stattic also lowered the expression and phosphorylation of p65 in cytosol and nuclei (Fig. 7B).

### Elevated P-gp in T-lymphocytes and monocytes affected the cellular pharmacokinetics of Cys A

**Figure 8:**
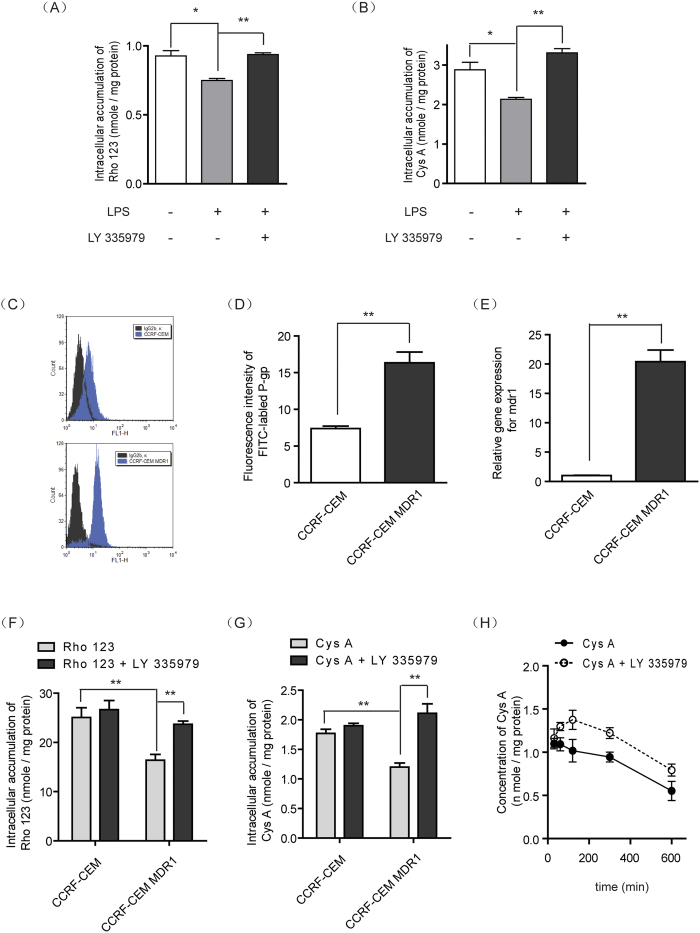
Elevated P-gp levels in monocytes and T-lymphocytes affected the intracellular pharmacokinetics of Cys A. THP-1 monocytes were pre-incubated with LPS for 72 h for P-gp induction, which was confirmed by an intracellular retention assay of the P-gp substrate probe Rho 123 (**A**), and the P-gp substrate immunosuppressant Cys A (**B**) in the presence or absence of the P-gp inhibitor LY335975. CCRF-CEM MDR1 lymphocytes were constructed from the CCRF-CEM parental cell line by lentiviral transfection. Their P-gp protein levels were analyzed by flow cytometry using a FITC-conjugated anti-P-gp antibody (**C**) and were quantified based on mean fluorescence intensity (**D**). The mRNA levels of P-gp were also measured by qPCR using actin as an internal control (**E**). The function of P-gp was evaluated through an intracellular retention assay using Rho 123 as a P-gp substrate probe (**F**) and Cys A as a P-gp substrate immunosuppressant (**G**) in the absence or presence of the P-gp inhibitor LY335979. (**H**) The uptake kinetics of the immunosuppressant Cys A in P-gp-overexpressing lymphocytes were determined by incubating CCRF-CEM MDR1 cells with Cys A in the absence or presence of the P-gp inhibitor LY335979. All of the data are expressed as the mean ± S.E.M.; *p < 0.05, **p < 0.01 compared with the corresponding control group; n = 3.

As seen in Fig 8A, inflammation-mediated P-gp-overexpressing monocytes were developed by incubating THP-1 cells with LPS. Rho 123 as an indicator of P-gp function; its intracellular accumulation was markedly decreased in LPS-treated THP-1 cells and could be reversed by the application of P-gp inhibitor. For the P-gp substrate drug Cys A, LPS-mediated inflammatory monocytes exhibited lower intracellular accumulation of Cys A, which was only 69% of that in the control cells and could be reversed by the application of P-gp inhibitor (Fig. 8B).

To further mimic the P-gp-overexpressing state of PBMC in colitis, human CCRF-CEM T-lymphocyte cells were transfected with P-gp via a lentiviral system, and the overexpression of P-gp was confirmed by flow cytometry assay (Fig. 8C) and qPCR assay, which demonstrated a 2.2-fold increase of protein expression (Fig. 8D) and a 20-fold increase of gene expression (Fig. 8E), respectively. Furthermore, an assay that tested P-gp function via the assessment of Rho 123 suggested a 35% decrease of P-gp intracellular accumulation (Fig. 8F). For the immunosuppressive agent Cys A, its cellular accumulation in CCRF-CEM MDR1 cells, which simulated PBMC in colitis, was significantly decreased by 33.4% and could be reversed by the application of P-gp inhibitor (Fig. 8G). The uptake kinetics of Cys A in CCRF-CEM MDR1 cells exhibited a time-dependent linear decrease. However, in the presence of P-gp inhibitor, the intracellular concentrations of Cys A were markedly elevated and presented a two-phase absorption: before 2 h post drug administration, the concentrations of Cys A increased with time, whereas after 2 h the concentrations of Cys A declined with the same elimination rate of the control group (Fig. 8H).

## Discussion

TNBS- and dextran sodium sulfate (DSS)-induced colitis are both well-established animal models for IBD studies^35^. Compared to DSS-induced colitis, TNBS-induced colitis displays a disorder of Th1 and Th17 with an enhanced intensity of Th1/Th17 response, which could simulate the characteristics of CD-related immune responses quite well^31^,^36^,^37^. In view of this, TNBS-induced colitis has been used in our laboratory to explore the metabolism and disposition features of drugs under a CD pathologic state. For example, our previous studies revealed an isoform-dependent and tissue-specific dysregulation of an important phase II metabolizing enzyme, UDP-glucuronosyltransferase (UGT), in TNBS-induced rats^38^. As metabolizing enzymes and drug transporters are two key factors for the disposition of immunosuppressive drugs *in vivo*, we continued to analyze drug transporters under a CD state in this study.

When the immunosuppressive drug Cys A was intravenously administered to TNBS-induced colitis rats, its plasma pharmacokinetics profiles changed significantly, with an increased elimination rate *k* and a decreased AUC, relative to those in normal rats (Fig. 1A). The target of Cys A is calcineurin, which induces T-cell activation^39^. Because the blood concentrations of Cys A in renal transplant recipients did not predict its effect on calcineurin activity^40^, and as Cys A monitoring in PBMC is more feasible and credible^41^, we further analyzed drug concentrations in PBMC and found that less Cys A entered into the PBMC of TNBS-induced rats when plasma drug concentrations were equivalent to those in normal rats and TNBS-induced colitis rats at the first two time points (Fig. 1B,C). These results suggested that the effective exposure of immunosuppressive drugs on target cells in CD might be lowered, which possibly led to compromised pharmacological effects and caused intrinsic resistance. Similar results were also obtained for another immunosuppressive drug, tacrolimus, in TNBS-induced colitis rats (Supplementary Fig 2).

To elucidate these results and analyze their potential mechanisms, we further selected BALB/c mice as a model animal, as they are more sensitive to TNBS and because their symptoms more closely resemble the features of CD^42^. At 3 days post TNBS administration, distinct inflammatory injury and infiltration of the colon were observed in these mice (Supplementary Fig 1), and some cytokines levels in peripheral plasma were highly increased (Fig. 4). With the prolongation of recovery time from 3 days to 7 days post TNBS challenge, the body weights of the model mice gradually recovered (Supplementary Fig 1A), and the severity level of TNBS-induced colitis was also relieved (Supplementary Fig 1D). During this subacute process, most inflammatory cytokines, such as TNF-α, IL-1β, IL-6 and LPS, in the blood were down-regulated, although they were still much higher than the corresponding levels in the control groups (Fig. 4).

It is interesting to note that P-gp expression in the PBMC of model mice remained unchanged on the 3^rd^ day post TNBS challenge, when inflammation symptoms were the most serious. Instead, P-gp was significantly activated in the PBMC of model mice on the 7^th^ day (Fig. 2), which was indicated by elevated mdr1a gene expression (Fig. 2A) and decreased intracellular accumulation of the P-gp substrate Rho 123 (Fig. 2B). When the commonly used immunosuppressive agent Cys A was applied to the PBMC of model mice, they were also extruded out of cells and therefore had lesser retention within cells (Fig. 2C). This indicated that there was distinct intrinsic drug resistance during the subacute phase but not during the acute phase. This phenomenon was also observed in DSS-induced colitis mice, as there was a 6.29-fold up-regulation in mdr1a gene expression in the PBMC of colitis mice on the 9^th^ day post DSS challenge (Supplementary Fig 3). Further flow cytometry analysis revealed the domination of T-lymphocytes with significantly higher P-gp expression in the PBMC of colitis mice (Fig. 3).

Thus far, we have hypothesized that chronic low-grade inflammation might not lead to lethal damage, but it may possibly influence the expression and function of some proteins, such as P-gp. For example, tissue eosinophilia and radiographic inflammation can up-regulate P-gp expression, and peripheral inflammatory pain can lead to specific structural changes in P-gp^43^,^44^. A change in P-gp expression has also been found in several autoimmune diseases, as well as in IBD^4^,^21^,^31^. With the development of autoimmune disease, cytokines become increasingly secreted, some of which in turn induce the expression of P-gp in immune cells^16^,^45^. Thus, we explored the effects of cytokines and LPS, which have been proven to be increased in the subacute model (Fig. 4), on P-gp expression in immune cells *in vitro*. CCRF-CEM is a type of human T-lymphocyte, and THP-1 is a type of human monocyte; both are the main constituents of PBMC that contribute to high P-gp expression in colitis. Our results indicated that TNF-α and IL-17 exhibited the most potent stimulatory effects on P-gp expression in CCRF-CEM cells, while LPS and TNF-α presented the most potent effects in THP-1 cells (Fig. 5).

To further elucidate the potential mechanisms for the induction of P-gp by cytokines and LPS in immune cells, the NF-κB pathway was focused on because it is highly related to chemo- and radio-resistance during cancer treatment^46^, and its key role in P-gp regulation has been demonstrated in our previous studies^33^. As anticipated, cytokine- and LPS-induced elevations in P-gp expression were all markedly reversed by the NF-κB inhibitor Bay 117082 in both cell types *in vitro* (Fig. 6), suggesting the involvement of the NF-κB pathway. Next, TNF-α time-dependently enhanced the translocation of p65 from cytosol into nuclei and the further phosphorylation of p65 in the nuclei of CCRF-CEM cells (Fig. 7A). In THP-1 cells, LPS and TNF-α could stimulate both p65 and phosphorylated p65 expression in cytosol and nuclei (Fig. 7A). These activations of p65 potentially led to higher expression of P-gp (Fig. 5).

As for IL-17, it was found to be continuously increasing through 7 days post TNBS challenge, which is obviously different from other cytokines and LPS (Fig. 4). This uninterrupted enhancement of IL-17 originated from the induction of IL-17A-expressing CD4^+^ T cells (Th17 cells) and is regarded as a key effector of mucosal inflammation in CD and in other inflammatory autoimmune disorders^47^,^48^. IL-17 acts mainly through the signal transducer and activation of transcription 3 (STAT3), which is a cytoplasmic protein that plays a pivotal role in the regulation of different types of immune and inflammatory responses^49^,^50^. In our experiment, applying the STAT3 inhibitor Stattic significantly attenuated P-gp up-regulation activated by IL-17 in both cell types *in vitro* (Fig. 6). In addition, it was found that IL-17 treatment promoted STAT3 expression and phosphorylation in a time-dependent manner (Fig. 7B). Meanwhile, the expression levels and phosphorylation of p65 both in cytosol and nuclei were also elevated, and this up-regulation could be reversed by the application of STAT3 inhibitor, which indicated that STAT3 was activated after phosphorylation and that it subsequently activated the Nf-κb pathway (Fig. 7B). Thus, the high levels of IL-17 secretion in CD may induce P-gp expression through the STAT3-dependent Nf-κb pathway and then lead to intrinsic resistance.

Such an enhancement of P-gp in immune cells by inflammatory induction might influence the intracellular disposition of immunosuppressive drugs and finally result in a compromised clinical therapeutic effect^21^. Therefore, we continued to mimic the P-gp-overexpressing immune cells that are observed in CD *in vitro* by either inflammatory stimulation or stable transfection (Fig. 8). In both models, the intracellular accumulation of Cys A was significantly lowered relative to normal cells. Meanwhile, following the co-administration of a P-gp inhibitor, the decreased Cys A concentrations in immune cells in CD status could effectively be reversed and restored to normal levels (Fig. 8A,B,F–H). Further kinetic analysis of Cys A in immune cells revealed the time course of P-gp regulation in the intracellular fate of Cys A (Fig. 8H) and indicated that P-gp inhibitor might be helpful for enhancing the effect of immunosuppressive drugs and overcoming intrinsic resistance in CD patients through P-gp regulation.

**Figure 9:**
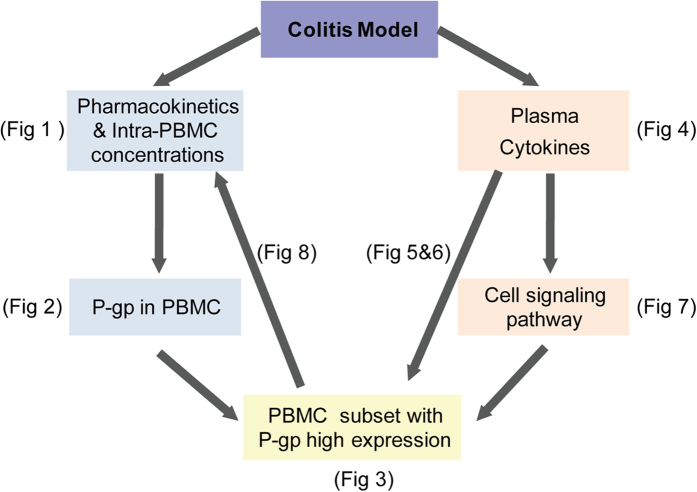
A schematic diagram of present studies from phenomena to mechanisms.

In conclusion, our work revealed decreased retention of Cys A in the PBMC of a TNBS-induced colitis animal model and suggested the possible mechanism that chronically elevated inflammation in blood could induce P-gp up-regulation in PBMC through the STAT3/Nf-κb pathway (Fig. 9). Based on our findings, it may be useful to evaluate P-gp levels in the PBMC of CD patients before they take medicine. Additionally, our work suggested that anti-inflammation could be taken prior to immunosuppressive agents, as anti-inflammation might block the induction of P-gp in PBMC and thus promote the uptake of immunosuppressants into PBMC and enable them to take action. Finally, combined usage of a P-gp inhibitor might be beneficial to overcoming intrinsic/acquired resistance in CD patients in the clinical.

## Additional Information

**How to cite this article**: Liu, J. *et al.* Chronic inflammation up-regulates P-gp in peripheral mononuclear blood cells via the STAT3/Nf-κb pathway in 2,4,6-trinitrobenzene sulfonic acid-induced colitis mice. *Sci. Rep.* **5**, 13558; doi: 10.1038/srep13558 (2015).

## Supplementary Material

Supplementary Information
