# Supplementary material for: Chronic inflammation up-regulates P-gp in peripheral mononuclear blood cells via the STAT3/Nf-κb pathway in 2,4,6-trinitrobenzene sulfonic acid-induced colitis mice
Source: Sci Rep. 2015 Sep 1;5:13558. doi: 10.1038/srep13558 (PMC4555107; doi:10.1038/srep13558)
Supplement: Supplementary Information [file srep13558-s1.pdf]

Chronic inflammation up-regulates P-gp in peripheral mononuclear blood cells via the STAT3/Nf-κb pathway in 2,4,6-trinitrobenzene sulfonic acid-induced colitis mice

Jiali Liu[1,2], Fang Zhou[1,2,3], Qianying Chen[1], An Kang[4,*], Meng Lu[1], Wenyue Liu[1], Xiaojie Zang[1], Guangji Wang[1,3,*], and Jingwei Zhang[1,3,*]

[1]Key Lab of Drug Metabolism and Pharmacokinetics, State Key Laboratory of Natural Medicines, China Pharmaceutical University, Nanjing, Jiangsu, China

[2] Jiali Liu and Fang Zhou contributed equally to this work.

[3]Jiangsu Key laboratory of drug design and optimization, China Pharmaceutical University, Nanjing, Jiangsu, China.

[4]School of Pharmacy, Nanjing University of Chinese Medicine, Nanjing, Jiangsu, China

* Corresponding author:

Professor Guangji Wang, Key Lab of Drug Metabolism and Pharmacokinetics, China Pharmaceutical University, 24 Tong Jia Xiang, Nanjing, Jiangsu, 210009, China. E-mail: guangjiwang@hotmail.com

Associate Professor Jingwei Zhang, Key Lab of Drug Metabolism and Pharmacokinetics, China Pharmaceutical University, 24 Tong Jia Xiang, Nanjing,

Jiangsu, 210009, China. E-mail: zhangjw_cnnj@sina.com

Lecturer An Kang, School of Pharmacy, Nanjing University of Chinese Medicine, 138 Xianlin Road, Nanjing, Jiangsu, 210023, China. E-mail: kanga@njucm.edu.cn

Supplementary Fig 1. Development of acute and subacute TNBS-induced mice. BALB/c mice were administered with TNBS (2.5 mg/0.1 ml/0.02 kg in 50% ethanol) or equal volume of 50% ethanol into the colons via a thin catheter. The body weight was monitored every day and weight changes were plotted against time (A). On the 3rd day or 7th day post vehicle or TNBS challenge, mice were sacrificed and specimens of proximal colon were collected, imaged and measured afterwards (B). After being fixed and embedded, sections of proximal colon were cut at a thickness of 5 μm, stained with H&E, and imaged with Leica microscope (C). The histological damage was evaluated and graded in a double-blinded fashion by a skilled pathologist (D). Data are expressed as the mean±S.E.M.. n=8/group.

Supplementary Fig 2. Plasma and cellular pharmacokinetics profiles of Tacrolimus in control and TNBS-induced rats. The control group was given blank vehicle intrarectally and the model group was administered intrarectally a dose of 100 mg/kg TNBS. On the 7th day, both groups were intravenously administered with Tacrolimus at the dosage of 0.5 mg/kg. Blood was collected at designated time-point post Tacrolimus administration, and plasma and PBMC were separated. Concentrations of Tacrolimus in plasma and PBMC were determined by LC-MS/MS, and concentration-time curves of Tacrolimus in plasma (A) and PBMC (B) were plotted. The AUC of Tacrolimus in plasma and PBMC were calculated using the trapezoidal method (C). The ratio of Tacrolimus concentration in PBMC to that in plasma at 30 min, 60 min and 90 min post Tacrolimus administration was also

calculated (D). Data are expressed as the mean±S.E.M.; n=5/group. * $p<0.05$, ** $p<0.01$ between model group versus corresponding control group.

Supplementary Fig 3. Development of DSS-induced colitis mice. C57BL/6 mice (8 week, 20-22g) were classified into two groups: mice in model group were administered of 3% (w/v) DSS in drinking water continuously from day 0 to day 5, and mice in control group received the same drinking water without DSS. From day 6 to day 9, mice in both groups received drinking water without DSS. The body weight was monitored on 0, 2, 4, 6, 8 and 9 day and weight changes were plotted against time (A). On the 9th day, mice were sacrificed and specimens of proximal colon were collected, imaged and measured afterwards (B). After being fixed and embedded, sections of proximal colon were cut at a thickness of 5 μm, stained with H&E, and imaged with Leica microscope (C). Furthermore, PBMC of mice in both groups were collected, and the mdr1a expressions in PBMC were determined by qPCR (D). Data are expressed as the mean±S.E.M.. n=8/group. *$p<0.05$ between the model group versus the corresponding control group.

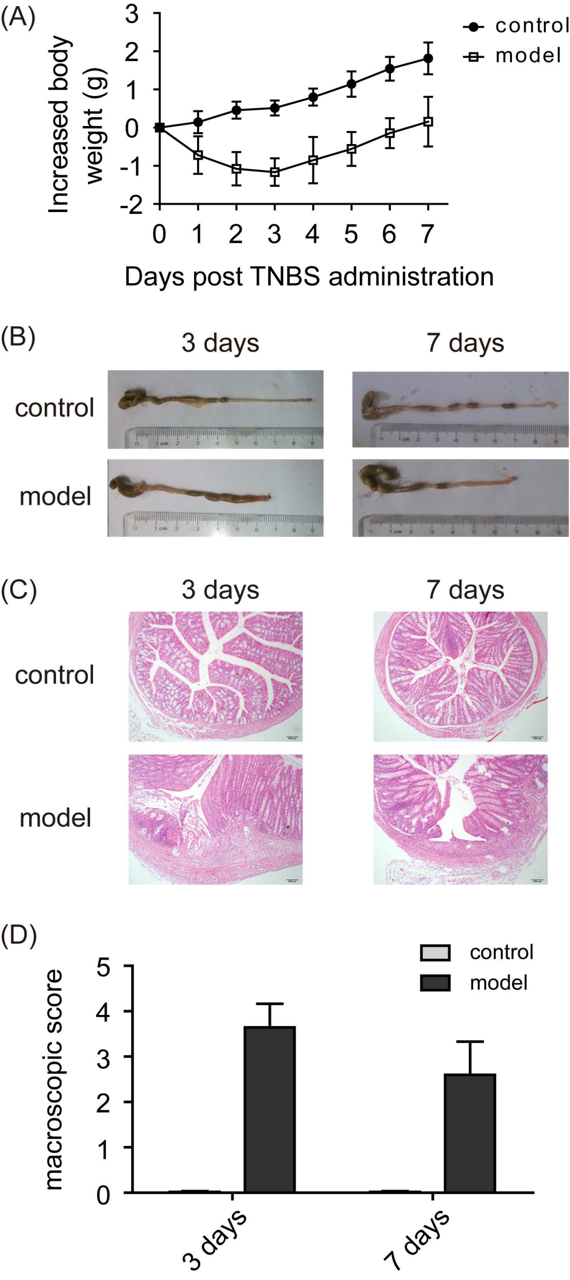
(A)
Increased body weight (g)
3
2
1
0
-1
-2
0 1 2 3 4 5 6 7
Days post TNBS administration
control
model
(B)
3 days
7 days
control
model
(C)
3 days
7 days
control
model
(D)
macroscopic score
5
4
3
2
1
0
3 days
7 days
control
model

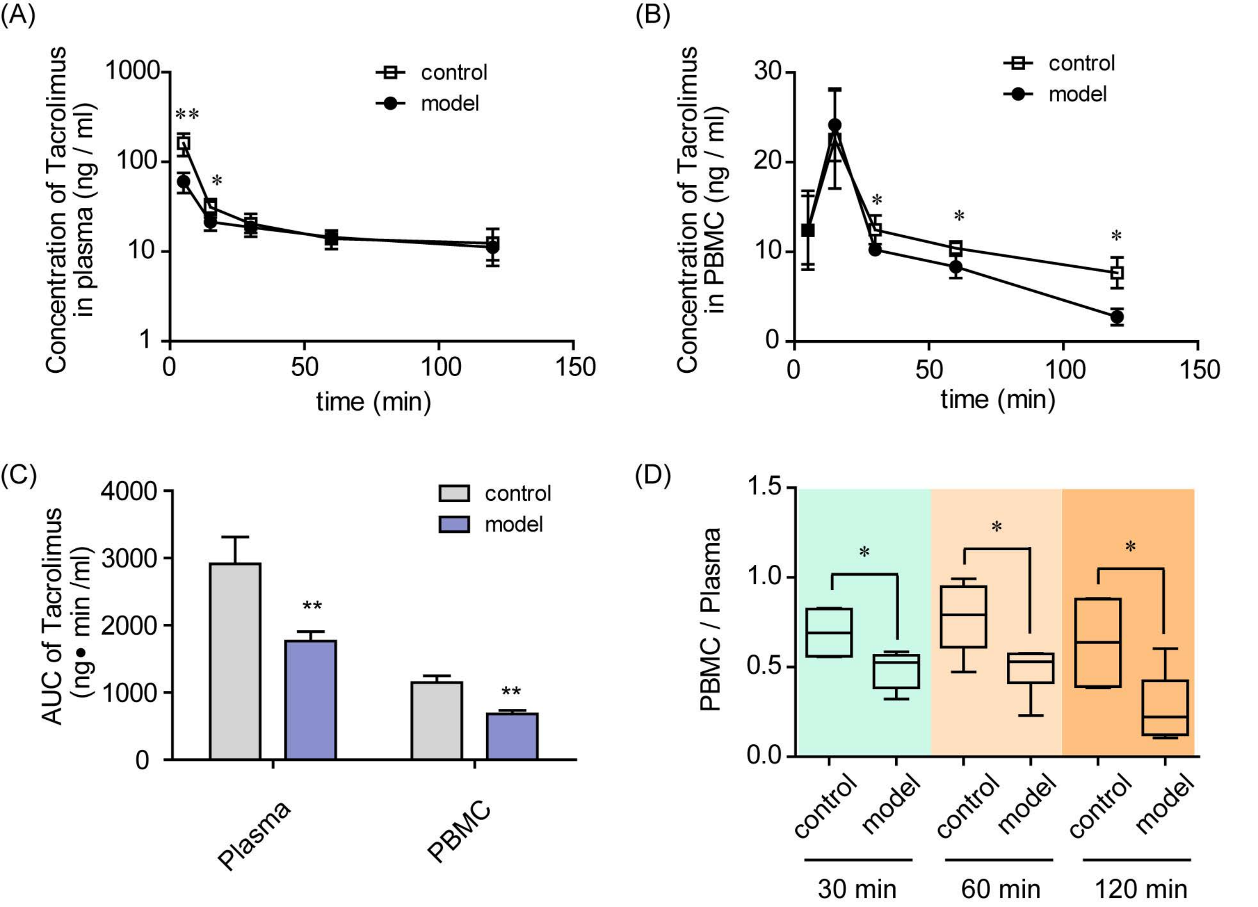

(A)
Concentration of Tacrolimus in plasma (ng / ml)
1000
100
10
1
0
50
100
150
time (min)
control
model
**
*
(B)
Concentration of Tacrolimus in PBMC (ng / ml)
30
20
10
0
0
50
100
150
time (min)
control
model
*
*
*
(C)
AUC of Tacrolimus (ng• min /ml)
4000
3000
2000
1000
0
control
model
**
**
Plasma
PBMC
(D)
PBMC / Plasma
1.5
1.0
0.5
0.0
*
*
*
control
model
control
model
control
model
30 min
60 min
120 min

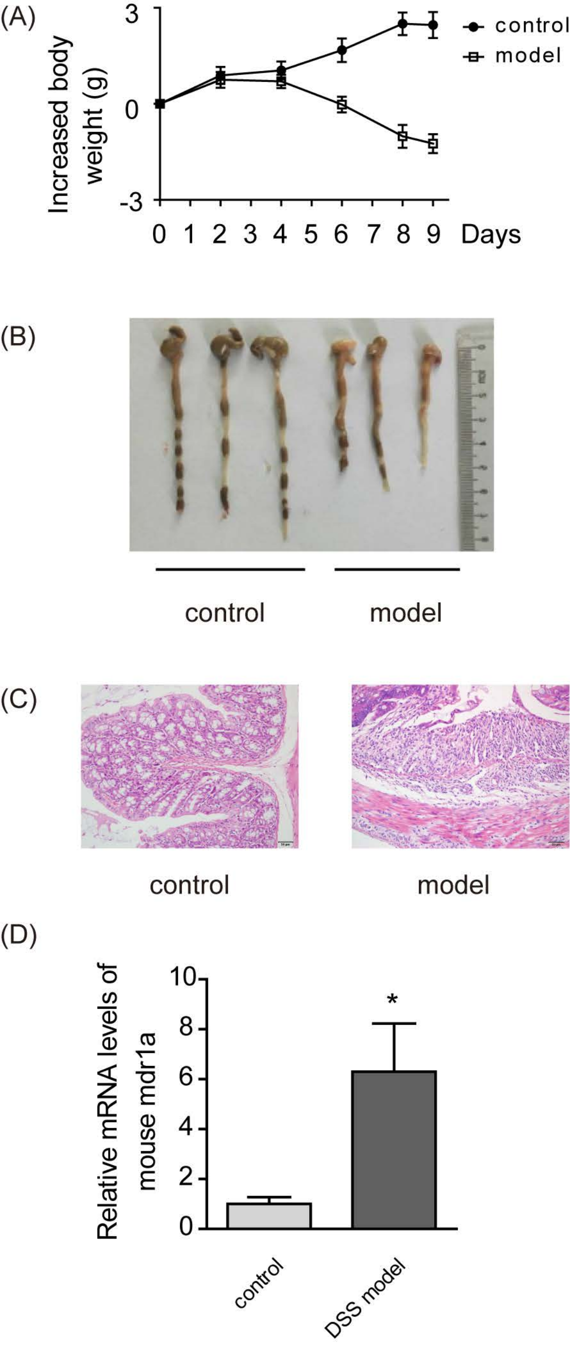
(A)
Increased body weight (g)
3
0
-3
0 1 2 3 4 5 6 7 8 9 Days
control
model
(B)
control
model
(C)
control
model
(D)
Relative mRNA levels of mouse mdr1a
10
8
6
4
2
0
*
control
DSS model
